# Cardiotoxicity Secondary to Immune Checkpoint Inhibitors in the Elderly: Safety in Real-World Data

**DOI:** 10.3390/cancers15174293

**Published:** 2023-08-28

**Authors:** Irene Toribio-García, Alejandro Olivares-Hernández, José Pablo Miramontes-González, Luis Posado Domínguez, Ana Martín García, Rocío Eiros Bachiller, Luis Figuero-Pérez, María Garijo Martínez, Jonnathan Roldán Ruiz, Lorena Bellido Hernández, Emilio Fonseca-Sánchez, Pedro Luis Sánchez, Edel del Barco-Morillo

**Affiliations:** 1 Department of Cardiology, University Hospital of Leon, 24008 León, Spain; itoribio@saludcastillayleon.es; 2 Department of Medical Oncology, University Hospital of Salamanca, 37007 Salamanca, Spain; lposadod@saludcastillayleon.es (L.P.D.); lfiguero@saludcastillayleon.es (L.F.-P.); mcgarijo@saludcastillayleon.es (M.G.M.); jroldanr@saludcastillayleon.es (J.R.R.); lbellido@saludcastillayleon.es (L.B.H.); efonseca@saludcastillayleon.es (E.F.-S.); 3 Institute for Biomedical Research of Salamanca (IBSAL), 37007 Salamanca, Spain; amartinga@saludcastillayleon.es (A.M.G.); reirosb@saludcastillayleon.es (R.E.B.); plsanchez@saludcastillayleon.es (P.L.S.); 4 Department of Internal Medicine, University Hospital Rio Hortega, 47012 Valladolid, Spain; jpmiramontes@hotmail.com; 5 Department of Medicine, University of Valladolid, 45005 Valladolid, Spain; 6 Department of Cardiology, University Hospital of Salamanca, 37007 Salamanca, Spain; 7 Department of Medicine, University of Salamanca, 37007 Salamanca, Spain

**Keywords:** immunotherapy, immune checkpoint inhibitors, elderly, cardiotoxicity, real-word data

## Abstract

**Simple Summary:**

Immunotherapy is the mainstay treatment for most solid tumours. However, its cardiotoxicity is not yet known and studied, and its uncertainty is even greater in elderly patients. For this reason, this ambispective study was conducted in 195 patients over 70 years of age who were treated with immunotherapy. The percentage of patients with cardiotoxicity was 1.54%; 1.35% of patients with previous heart disease were diagnosed with cardiotoxicity, and 1.65% of those without previous heart disease were diagnosed with cardiotoxicity. The median time from the initiation of treatment until the cardiac event was 45 days. The most frequent toxicity was myocarditis, which was identified in 66.7% of patients, followed by arrhythmias in 33.3% of patients. In conclusion, immunotherapy is shown to be a safe treatment in elderly cancer patients in terms of cardiotoxicity.

**Abstract:**

Introduction: Immunotherapy represents a key pillar of cancer treatments, with high response rates and long survival. Its use is increasing, mainly at the expense of the geriatric population due to the ageing of this population. However, despite its benefit, its safety in certain areas such as cardiotoxicity is largely unknown. The aim of this study is to assess the safety of immunotherapy in elderly patients using real-world data. Methods: This is an ambispective study of patients ≥ 70 years old with solid tumours who were treated with immunotherapy at the University Hospital of Salamanca. Cardiotoxicity was assessed using the CTCAEv5.0 criteria. Results: In total, 195 patients were included (76.9% male and 23.1% female), with a mean age of 75 years [70–93]. The percentage of patients with cardiotoxicity was 1.54%; 1.35% of patients with previous heart disease were diagnosed with cardiotoxicity, and 1.65% of those without previous heart disease were diagnosed with cardiotoxicity. The median time from the initiation of treatment until the cardiac event was 45 days [14–96]. The most frequent toxicity was myocarditis in 66.7% of patients, followed by arrhythmias in 33.3% of patients. Conclusions: Immunotherapy is shown to be a safe treatment in elderly cancer patients in terms of cardiotoxicity. The event rate shows no difference between patients with or without cardiac comorbidity.

## 1. Introduction

Immunotherapy based on immune checkpoint inhibitors (ICIs) has been a revolution in the treatment of cancer patients which has experienced exponential growth over the last 10 years [1]. Since the approval of Ipilimumab (an antibody against the CTLA-4 receptor) for the treatment of metastatic melanoma [2], several drugs have been introduced into solid tumour treatment regimens (Supplementary Material, Table S1) [3,4]. The rates of patients alive at 5 years with metastatic tumours who are treated with immunotherapy for lung cancer or melanoma are as high as 20–30% [5]. Furthermore, these drugs are increasingly used in patients over 70–75 years of age due to the ageing of the Western population [6].

However, their increased use in routine clinical practice, in many cases in patients belonging to the geriatric population, also leads to an increase in adverse side effects. These immuno-related effects (irAEs) can affect virtually any organ, and in rare cases, the consequences can be severe (1–2% mortality) [7]. The iatrogenicity described is very diverse, but one of the most serious and currently underestimated complications is cardiovascular toxicity [8].

**Figure 1:**
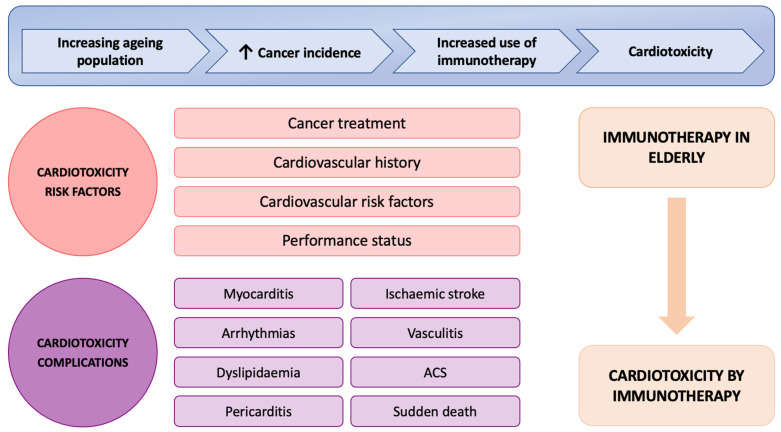
Illustration of the causes and risk factors for cardiotoxicity secondary to immunotherapy in an elderly patient. The ageing of the population coupled with the increased development of ICI therapies in solid tumours has led to an increased occurrence of immuno-related adverse effects including cardiotoxicity.

Immune-related cardiovascular adverse events comprise a wide range of entities, the most frequent being arrhythmias, myocarditis with or without ventricular dysfunction (symptomatic or asymptomatic), and pericardial abnormalities (Figure 1) [9]. Cardiotoxicity associated with ICIs is estimated to have an incidence of 1–3%, although more recent studies have shown rates as high as 10–12% [10,11,12], although it varies between series, as well as according to the treatments prescribed (the rate is higher in combination treatments of anti-PD(L)1 with anti-CTLA4) [13]. The morbidity and mortality that this toxicity can cause in cancer patients, especially in the geriatric population, makes it essential to know the safety of immunotherapy in this spectrum of patients. Furthermore, cardiotoxicity presents a wide spectrum of clinical manifestations that are currently under study which are even more complex to analyse in the geriatric population. The aim of this study is to assess the safety of immunotherapy treatments on the occurrence of cardiovascular events in elderly cancer patients using real-life data in solid tumours.

## 2. Materials and Methods

### 2.1. Sources of Data

An ambispective study was performed with a population registered through the data system of the Complejo Asistencial Universitario de Salamanca (Salamanca, Spain). The patients selected for this study were those with a diagnosis of a solid tumour who had received immunotherapy treatment (anti-PD1, anti-PDL1, or anti-CTLA4) with an age at the start of treatment greater than or equal to 70 years. Data on drug use, as well as clinical and analytical data, were obtained from the medical records and databases of the Medical Oncology Service.

The fundamental data collected for this study were:

- Epidemiological: age (years) and sex.
- Oncological: tumour (location, stage, histology, and oncological treatment), PDL-1, and driver mutations.
- Type of treatment: type of immunotherapy, number of doses, and line treatment.
- Response: overall survival (OS), progression-free survival (PFS), overall response rate (ORR), and disease control rate (DCR).
- Cardiac: cardiac comorbidity, the treatment of cardiac pathology, and cardiotoxicity (number and types of events).
- Clinical and analytical data and comorbidities: general condition (Eastern Cooperative Oncology Group, ECOG), analytical data (blood count, creatinine, liver function, proteins, and glycemia), body mass index (BMI), general toxicity (grade), and associated cardiovascular factors and comorbidities.

The patients selected for the study were treated at the Complejo Asistencial Universitario de Salamanca in all cases, and their treatments were followed until the time of data analysis.

### 2.2. Cohort Construction

The cohort of patients for the study was established via hospital discharge records collected between 1 January 2014 and 31 August 2022 (Supplementary Material, Figure S1). The prospective cohort was recruited from January 2021. The cohort was restricted to those individuals who were diagnosed with a metastatic solid tumour and treated with immunotherapy at an age equal or greater than 70 years. To facilitate the analysis, two baseline cohorts were also created according to the types of tumours the patients had (subgroup 1 comprised patients with non-small-cell lung cancer “NSCLC”, and subgroup 2 comprised patients with all other solid tumours). Because there is no consensus in oncology to define at what age a patient belongs to the geriatric population, 70 years was chosen as the cut-off age in most studies in this field (American Society of Clinical Oncology “ASCO” definition).

#### 2.2.1. Inclusion Criteria

- Patients who had received ICIs in any line of treatment, both in monotherapy and in different combinations such as with chemotherapy, other ICIs, or tyrosine kinase inhibitors.
- Patients with a diagnosis of a solid tumour in any stage.
- Patients who had all the clinical information necessary for the analysis of the objectives collected.

#### 2.2.2. Exclusion Criteria

- Patients using immunotherapy for haematological tumours.
- The clinical information necessary for the study had not been collected.

### 2.3. Objectives and Definitions

The primary objective of this study was to assess the safety of immunotherapy treatments in elderly cancer patients on the occurrence of cardiac events in the context of cardiotoxicity (defined as a cardiac event or alteration secondary directly or indirectly to immunotherapy treatment). Immune-related cardiac toxicity was established and defined in patients using the definitions of the main clinical guidelines. The secondary objectives were to analyse whether the safety of immunotherapy is similar in patients who have cardiac comorbidities versus those who do not and to study the epidemiology of the cohort to understand the phenotype of geriatric oncology patients with cardiac comorbidity in whom immunotherapy is to be used.

Cardiac comorbidity was grouped into five subgroups (arrhythmic, cardiomyopathies and pericardial abnormalities, ischaemic, valvular heart disease, and heart failure). Cardiac events were subdivided into those related to immunotherapy versus those categorised as not related to immunotherapy. PFS was defined as the time in months from the initiation of immunotherapy treatment to radiological or clinical progression, patient death, or loss to follow-up during treatment. OS was the time in months from the initiation of immunotherapy treatment to patient death or loss to follow-up. ORR was defined as the proportion of patients whose best radiological response was a complete response (CR) or partial response (PR), and DCR was the percentage of the sum of CR, PR, and disease stabilisation (DS).

Radiological response was established in four groups (CR, PR, SD, progression, or PD), according to the definition of the Response Evaluation Criteria in Solid Tumors, version 1.1 (RECIST v1.1). Toxicity was collected and graded according to the Common Terminology Criteria for Adverse Events (CTCAE v5.0). For cardiotoxicity, CTCAE v5.0—27 November 2017, pages 5–10, was used in June 2023 (available at: https://ctep.cancer.gov/protocoldevelopment/electronic_applications/docs/ctcae_v5_quick_reference_8.5x11.pdf (accessed on 1 December 2017)).

### 2.4. Statistical Analysis

Estimates of the differences between populations at the epidemiological level were estimated using comparative statistical tests of medians and percentages. Survival rates were calculated in months and expressed as medians with 95% confidence intervals (CIs). Median survival rates were calculated using the Kaplan–Meier method and compared using log-rank tests. Univariate and multivariate Cox proportional hazards models were constructed to test the association of predictive factors with PFS and OS.

To avoid confounding factors, sub-analyses were established. Additional proportional hazards models were constructed including the potential confounders. In the results section, lung cancer patients were analysed in a separate subgroup as a majority subpopulation. A logistic regression model was constructed to explain those factors explaining response or poor response to ICIs. The statistical significance of the study was established a priori at *p* < 0.05. The software used was SPSS, version 25 (IBM^®^).

## 3. Results

### 3.1. General Characteristics of the Sample

A total of 195 elderly patients treated with immunotherapy for metastatic solid tumours were included for the study (general characteristics of the sample are given in Table 1). In total, 76.9% of the patients were males and 23.1% were females. The mean age of the study was 75 years [70–93]. The most frequent tumour was represented by NSCLC, with 99 patients out of 195 (50.8%), followed by urothelial carcinomas with 29 patients (14.9%), melanoma with 23 patients (11.8%), renal tumours with 22 patients (11.3%), and small-cell lung carcinoma (SCLC) with 7 patients (3.6%). The most commonly used drug of all was Nivolumab in 94 patients (48.2%), followed by Pembrolizumab in 64 patients (32.8%), and Atezolizumab in 27 patients (13.8%). The mean number of doses received per patient was 7.5 doses [1–98].

When analysing the overall immunotoxicity of the sample, 56 (28.7%) of the total number of patients had some type of immuno-related toxicity. The most frequently observed toxicity was thyroiditis in 14 patients (7.2%), followed by gastrointestinal toxicity in 9 patients (4.6%) and hepatic toxicity in 9 patients (4.6%). Toxicity was grade 3–4 in 15 patients (7.7%). Within grade 4 toxicity (5 patients, 2.1%), haematological toxicity was observed in two patients (neutropenia in one patient and autoimmune thrombopenia in another patient). The remaining grade 4 toxicities were pneumonitis, nephritis, and colitis (see Table 2).

**Figure 2:**
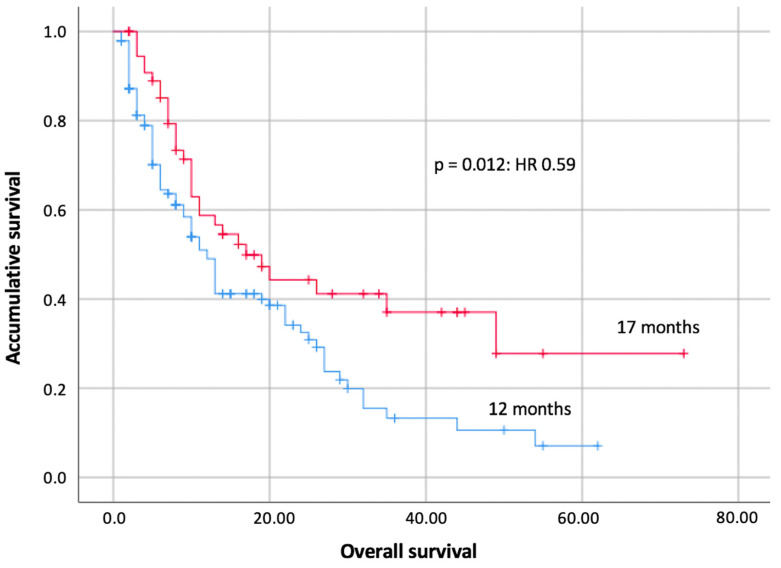
Overall survival compared between patients with toxicity versus those without toxicity. The red curve represents patients who had immunotoxicity versus the blue curve for those who did not. The result is statistically significant with a *p* = 0.012.

There was a statistically significant association between OS and those patients with some form of immunotoxicity. OS was observed for 17 months in those patients with immunotoxicity versus 12 months in those without (*p* = 0.012; HR 0.59 [95 CI 0.39–0.91]) (Figure 2). This association was not observed for PFS, which was 10 months for patients with immunotoxicity versus 7 months for patients without immunotoxicity (*p* = 0.151). A statistically significant association was also observed between response and toxicity (*p* = 0.004). Of the total of nine complete responses in the sample, a total of seven had some form of immunotoxicity.

### 3.2. Cardiac Comorbidity of the Sample

Within the majority subgroup of patients with NSCLC, a total of 35 patients out of 99 (35.4%) had some type of cardiac comorbidity, with some patients having more than one cardiac comorbidity. Within the classification groups, the comorbidities presented were as follows: (1) arrhythmias, 14 patients (14.1%); (2) ischaemic pathology, 15 patients (15.2%); (3) heart failure, 6 patients (6.1%); (4) valvular heart disease, 5 patients (5.1%); and (5) cardiomyopathies and pericardial abnormalities, 2 patients (2.0%).

In the rest of the tumour group, 39 patients out of 96 (40.6%) had some type of cardiac comorbidity prior to the initiation of immunotherapy treatment. The following comorbidities were found: (1) arrhythmias, 6 patients (6.3%); (2) ischaemic pathology, 15 patients (15.6%); (3) heart failure, 4 patients (4.2%); (4) valvular heart disease, 2 patients (2.1%); and (5) cardiomyopathies and pericardial abnormalities, 2 patients (2.1%).

In addition to cardiac comorbidity, the presence of cardiovascular risk factors in the sample and the patients’ ECOG data at the start of immunotherapy treatment were analysed. A total of 49 patients had no cardiovascular risk factors at the initiation of ICI (25.1%), with 46 patients having diabetes mellitus (23.6%), 102 patients having hypertension (52.3%), and 100 patients having dyslipidaemia (51.3%). The ECOG scores of the included patients were, in most cases, 0–1 (164 patients, 84.1%), and ≥2 in 31 patients (15.9%).

### 3.3. Cardiac Events Post-Immunotherapy (Primary Endpoint)

Among the patients with NSCLC, 15 patients had a post-immunotherapy cardiac event (15.2%), with a total of 6 patients having arrhythmic events, 1 patient having an alteration derived from valvular heart disease, 6 patients having heart failure or the decompensation of already diagnosed heart failure, and 4 patients having cardiomyopathy or alterations of the pericardium. Of the 64 patients without previous cardiac comorbidity, a total of 5 patients had a de novo cardiac event (7.8%). Among the 35 patients with previous cardiac pathologies, a total of 10 patients had cardiac episodes (28.6%). Of all the events observed in the 99 patients, only one (1.01%) was associated with immunotoxicity (atrial tachycardia in the context of second-line nivolumab monotherapy) in a patient with no history of heart disease.

Of the remaining patients in the sample, 10 patients had a cardiac event (10.5%). Arrhythmic events were observed in five patients, valvular heart disease in two patients, de novo heart failure or decompensation in two patients, and cardiomyopathy or pericardial abnormalities in three patients. The number of patients with events among the group with previous heart disease was five (12.8%). Among patients without heart disease, the number of patients who had an episode was four (7.02%). There were two immunotherapy-related events (2.1%). One event was grade 5 myocarditis (death of the patient) in a patient without previous heart disease (treatment with nivolumab monotherapy), and the other event was grade 2 myocarditis in a patient with previous heart disease (treatment with pembrolizumab in combination with an ITK).

Thus, in the total sample of 195 patients, there were a total of three immuno-related cardiac events (1.54%). Among patients with previous heart disease (74 patients) the number of cardiotoxic events was one (1.35%). Among patients without heart disease (121 patients) the number of patients with immuno-related events was two (1.65%). The median number of days from the start of immunotherapy to the cardiac event was 45 days [14–96]. A summary is provided in Table 3.

## 4. Discussion

Nowadays, immunotherapy treatments are increasingly studied and known in clinical practice [14]. Immune-related side effects and treatments for these complications are well detailed in clinical guidelines [15]. However, cardiotoxicity is one of the most common morbidity and mortality issues, with even greater implications in patients belonging to the geriatric population [16]. As indicated in the introductory section, cardiotoxicity is estimated to range from 1 to 3% (from 10 to 12% in several series), although its incidence and severity vary greatly depending on the type of cardiac alteration [17,18]. In the case of the study sample, the incidence of cardiotoxicity was 1.54%, which indicates that in global terms, cardiotoxicity in the spectrum of geriatric patients is similar to the series published so far [19,20,21,22].

### 4.1. Submission Times of Cardiotoxicity

A second important fact to understand the lack of data on cardiotoxicity as an irAE is the time to the onset of the event. In the different series, the range varies greatly, with the median number of days in the different studies ranging from 17 to 65 days [23]. Among the patients included in our study who experienced toxicity, the median day was 45 days, reflecting along with the incidence the similarity of cardiotoxicity in the geriatric patients in our investigation, along with the data at the general level. In all cases of cardiotoxicity, the treatment was an anti-PD1 without combination with another ICI, although only three patients out of 195 had combination treatment [24,25].

### 4.2. Cardiotoxicity in the Patients of the Sample (Secondary Objectives)

Among the types of cardiotoxicities, the most frequent and recognised is myocarditis, which accounts for 60–80% (0.1–0.2% overall as an irAE), followed by arrhythmias in 25–35% and pericardial alterations in 10–15% [16,26]. In our data, myocarditis accounted for 67% of all cardiac events and arrhythmias 33.3%. This figure is like the published figure, although there is a bias in both our series and the literature data secondary to the absence of data on the relationship between other cardiac pathologies and immunotherapy. It is therefore possible that there is an under-diagnosis of other events such as pericarditis or heart failure.

Another key finding in our sample was the division of the overall cohort between patients with and without a prior cardiac comorbidity [27]. Immune-related cardiovascular adverse events were similar between the two groups (1.35% vs. 1.65%, respectively). This shows that despite the overall safety in geriatric oncology patients, in whom incidence data are similar to the general population, there is no increased risk of a toxic event in those patients with a cardiac comorbidity [28,29]. This is of relevance in this population subgroup due to the high incidence of cardiac comorbidities compared to the general population in which younger patients have no previous cardiac disorders [30].

### 4.3. Limitations of the Study

After the interpretation of the data, it is important to be aware that the study has a number of limitations due to the fact that it was an ambispective study. In addition, the interpretation of cardiotoxicity in elderly patients is more complex due to the higher prevalence and incidence of cardiac pathology compared to the general oncology population. Furthermore, almost all patients received anti-PD(L)1 treatments without combinations with other immunotherapy drugs, so this may contribute to a bias in the results. The presence of these limitations, together with the absence of studies in this population, points to the need for prospective studies in this field.

## 5. Conclusions

Treatment with immune checkpoint inhibitors is well tolerated in elderly patients, as supported by the results of our study, which indicate a cardiotoxicity rate comparable to that observed in the general population. Moreover, in line with our results, the presence of cardiovascular comorbidities does not seem to influence the risk of immune-related cardiovascular adverse events. Further prospective studies will be needed in the future to validate these findings.

## Figures and Tables

**Table 1:** General characteristics of the patients in the sample.

Sample	NSCLC (*n* = 99)	Urothelial (*n* = 29)	Melanoma (*n* = 23)	Renal (*n* = 22)	Miscellany (*n* = 22)
Age (range)	74 (70–86)	76 (70–88)	78 (70–93)	76 (70–87)	76 (70–84)
Sex (M/W)	86/13 (86.9/14.1%)	24/5 (82.8/17.2%)	11/12 (47.8/52.2%)	15/7 (68.2/31.8%)	14/8 (63.6/36.4%)
Type of ICI Pembrolizumab Nivolumab Atezolizumab Nivo-Ipilimumab Ipilimumab Avelumab	48 (48.5%) 51 (51.5%) 0 (0%) 0 (0%) 0 (0%) 0 (0%)	4 (13.8%) 0 (0%) 20 (69%) 0 (0%) 0 (0%) 5 (17.2%)	0 (0%) 22 (95.7%) 0 (0%) 1 (4.3%) 5 (21.7%) 0 (0%)	4 (18.2%) 16 (72.8%) 0 (0%) 2 (9.1%) 0 (0%) 0 (0%)	8 (36.4%) 5 (22.7%) 7 (31.8%) 0 (0%) 0 (0%) 2 (9.1%)
Most common histology	ADC 62 (62.6%)	Transitional 27 (93.1%)	Superficial spreading 10 (%)	Clear cell 18 (81.8%)	SCLC 7 (31.8%)
Median doses of ICIs	9	6	8	6	6
ECOG 0–1 ≥2	83 (83.8%) 16 (16.2%)	25 (86.2%) 4 (13.8%)	20 (87%) 3 (13%)	19 (86.4%) 3 (13.6%)	17 (77.3%) 5 (22.7%)
Hypertension	52 (52.6%)	15 (51.7%)	17 (73.9%)	15 (68.2%)	13 (59.1%)
Dyslipidaemia	54 (54.5%)	16 (55.2%)	11 (47.8%)	8 (36.4%)	11 (50%)
Type 2 diabetes mellitus	22 (22.2%)	8 (28.6%)	7 (30.4%)	6 (27.3%)	3 (13.6%)
Cardiac comorbidity	39 (39.4%)	9 (31%)	7 (30.4%)	6 (27.3%)	9 (40.9%)

**Table 2:** Immunotoxicity observed in patients treated with ICIs in the study.

Immunotoxicity	Global (*n* = 195)	NSCLC (*n* = 99)	Urothelial (*n* = 29)	Melanoma (*n* = 23)	Renal (*n* = 22)	Miscellany (*n* = 22)
Global Grade 1–2 Grade 3–4	56 (28.7%) 41 (21%) 15 (7.7%)	25 (25.3%) 19 (19.2%) 6 (6.1%)	8 (27.6%) 7 (24.1%) 1 (3.4%)	11 (47.8%) 5 (21.7%) 6 (26.1%)	4 (18.2%) 4 (18.2%) 0 (0%)	8 (36.4%) 6 (27.3%) 2 (9.1%)
Endocrine Grade 1–2 Grade 3–4	14 (7.2%) 14 (7.2%) 0 (0%)	8 (8.1%) 8 (8.1%) 0 (0%)	2 (6.9%) 2 (6.9%) 0 (0%)	1 (4.3%) 1 (4.3%) 0 (0%)	0 (0%) 0 (0%) 0 (0%)	3 (13.6%) 3 (13.6%) 0 (0%)
Gastrointestinal Grade 1–2 Grade 3–4	8 (4.1%) 5 (2.6%) 3 (1.5%)	3 (3%) 1 (1%) 2 (2%)	2 (6.9%) 2 (6.9%) 0 (0%)	2 (8.7%) 1 (4.3%) 1 (4.3%)	0 (0%) 0 (0%) 0 (0%)	1 (4.5%) 1 (4.5%) 0 (0%)
Hepatic Grade 1–2 Grade 3–4	9 (4.6%) 4 (2.1%) 5 (2.6%)	4 (4%) 2 (2%) 2 (2%)	1 (3.4%) 1 (3.4%) 0 (0%)	2 (8.7%) 0 (0%) 2 (8.7%)	1 (4.5%) 1 (4.5%) 0 (0%)	1 (4.5%) 0 (0%) 1 (4.5%)
Asthenia Grade 1–2 Grade 3–4	6 (3.1%) 6 (3.1%) 0 (0%)	2 (2%) 2 (2%) 0 (0%)	1 (3.4%) 1 (3.4%) 0 (0%)	1 (4.3%) 1 (4.3%) 0 (0%)	0 (0%) 0 (0%) 0 (0%)	2 (9.1%) 2 (9.1%) 0 (0%)
Rheumatic Grade 1–2 Grade 3–4	4 (2.1%) 4 (2.1%) 0 (0%)	2 (2%) 2 (2%) 0 (0%)	0 (0%) 0 (0%) 0 (0%)	2 (8.7%) 2 (8.7%) 0 (0%)	0 (0%) 0 (0%) 0 (0%)	0 (0%) 0 (0%) 0 (0%)
Dermal Grade 1–2 Grade 3–4	4 (2.1%) 4 (2.1%) 0 (0%)	2 (2%) 2 (2%) 0 (0%)	0 (0%) 0 (0%) 0 (0%)	0 (0%) 0 (0%) 0 (0%)	1 (4.5%) 1 (4.5%) 0 (0%)	1 (4.5%) 1 (4.5%) 0 (0%)
Renal Grade 1–2 Grade 3–4	7 (3.6%) 4 (2.1%) 3 (1.5%)	5 (5.1%) 3 (3%) 2 (2%)	0 (0%) 0 (0%) 0 (0%)	1 (4.3%) 0 (0%) 1 (4.3%)	1 (4.5%) 1 (4.5%) 0 (0%)	0 (0%) 0 (0%) 0 (0%)
Pulmonary Grade 1–2 Grade 3–4	5 (2.6%) 2 (1%) 3 (1.5%)	2 (2%) 1 (1%) 1 (1%)	1 (3.4%) 0 (0%) 1 (3.4%)	0 (0%) 0 (0%) 0 (0%)	1 (4.5%) 1 (4.5%) 0 (0%)	1 (4.5%) 0 (0%) 1 (4.5%)
Haematological Grade 1–2 Grade 3–4	3 (1.5%) 1 (0.5%) 2 (1%)	0 (0%) 0 (0%) 0 (0%)	1 (3.4%) 1 (3.4%) 0 (0%)	2 (8.7%) 0 (0%) 2 (8.7%)	0 (0%) 0 (0%) 0 (0%)	0 (0%) 0 (0%) 0 (0%)
Muscular Grade 1–2 Grade 3–4	1 (0.5%)0 (0%)1 (0.5%)	1 (1%) 0 (0%) 1 (1%)	0 (0%) 0 (0%) 0 (0%)	0 (0%) 0 (0%) 0 (0%)	0 (0%) 0 (0%) 0 (0%)	0 (0%) 0 (0%) 0 (0%)

**Table 3:** Summary of cardiac events associated with ICIs following the initiation of immunotherapy treatment.

Cluster Sample (*n* = 195)	Global Cardiotoxicity	Myocarditis	Arrhythmias	Ischaemic	Heart Failure	Cardiomyopathies and Pericardial
Without cardiac comorbidity (*n* = 121)	2 (1.65%)	1 (0.82%)	1 (0.82%)	0 (0%)	0 (0%)	0 (0%)
With cardiac comorbidity (*n* = 74)	1 (1.35%)	1 (1.35%)	0 (0%)	0 (0%)	0 (0%)	0 (0%)
All patients (*n* = 195)	3 (1.54%)	2 (1.03%)	1 (0.51%)	0 (0%)	0 (0%)	0 (0%)

## Data Availability

The data included in the article can be found at the Department of Medical Oncology of the University Hospital of Salamanca.

## Supplementary Materials

The following supporting information can be downloaded at: https://www.mdpi.com/article/10.3390/cancers15174293/s1. Table S1: Main immune checkpoints antibodies approved for the treatment of solid tumours. Figure S1: Flow diagram of the study.
