# Supplementary material for: Cardiotoxicity Secondary to Immune Checkpoint Inhibitors in the Elderly: Safety in Real-World Data
Source: Cancers (Basel). 2023 Aug 28;15(17):4293. doi: 10.3390/cancers15174293 (PMC10486692; doi:10.3390/cancers15174293)

Table S1. - Main immune checkpoints antibodies approved for the treatment of solid tumours.

Immune checkpoint inhibitors	Immunoglobulin type	Target molecule	Tumour type
**Ipilimumab (MDX-010)**	IgG-1κ	CTLA-4	Advanced melanoma (alone second-line or in combination with Nivolumab in first-line).<br>Advanced renal cancer (in combination with Nivolumab in first-line).<br>Advanced lung cancer (in combination with chemotherapy and Nivolumab in first-line).<br>Advanced pleural mesothelioma (in combination with Nivolumab in first-line).<br>Advanced dMMR colorectal cancer (in combination with Nivolumab in first-line).<br>Advanced squamous oesophageal cancer (in combination with Nivolumab with PDL-1 ≥1%).
**Pembrolizumab (MK-3475)**	IgG-4κ	PD-1	Advanced melanoma and adjuvant (alone).<br>First-line metastatic NSCLC (alone in first PDL-1 ≥50% or in combination with CT in PDL-1 <50%).<br>Second line metastatic NSCLC (alone with PDL-1 ≥ 1%).<br>Refractory Hodgkin lymphoma (alone).<br>Advanced bladder cancer (alone in second-line).<br>Advanced head and neck cancer (in first line in combination with CT in CPS ≥ 1 or monotherapy in CPS ≥ 50%).<br>Advanced renal cancer (in combination with Lenvatinib or Axitinib) or in adjuvant.<br>All dMMR solid tumours (alone in first-line).<br>Advanced oesophageal cancer (plus CT in CPS ≥10%).<br>Advanced triple negative breast cancer (plus CT in CPS ≥10% or PDL-1+).<br>Neoadjuvant and adjuvant triple negative breast cancer (plus CT).<br>Advanced endometrial cancer (second-line with Lenvatinib).<br>Advanced cervical cancer (first-line with CT +/- Bevacizumab in CPS ≥1).
**Nivolumab (MDX-1106)**	IgG4	PD-1	Advanced melanoma (plus CT or alone) and adjuvant (alone).<br>First-line metastatic NSCLC (plus Nivolumab and CT).<br>Second-line metastatic NSCLC (alone).<br>Advanced pleural mesothelioma (in combination with Ipilimumab).<br>Advanced renal cancer (first-line with Ipilimumab or Cabozantinib and second-line alone).<br>Refractory Hodgkin lymphoma (alone).<br>Advanced bladder cancer (alone in second-line).<br>Advanced head and neck cancer (alone in second-line).

			Advanced dMMR colorectal cancer (in combination with Ipilimumab).<br>Advanced oesophageal cancer (plus CT or alone or plus Ipilimumab).
**Atezolizumab (MPDL3280A)**	IgG1	PD-L1	Advanced bladder cancer (alone in second line).<br>Metastatic NSCLC (in first-line in combination with CT and Bevacizumab or in second-line alone).<br>Advanced SCLC (in first-line plus CT).
**Durvalumab (MEDI4736)**	IgG1	PD-L1	Locally advanced unresectable non-small cell lung cancer (after CT/RT with PDL-1 ≥1% in tumour cells).<br>Advanced SCLC (in first-line plus CT).<br>Advanced hepatocellular carcinoma (in first-line in combination with Tremelimumab).
**Avelumab (MSB0010718C)**	IgG1	PD-L1	Metastatic Merkel cell carcinoma (alone).<br>Advanced urothelial cancer in maintenance after CT in first-line.<br>Advanced renal cancer (plus Axitinib in first-line).
**Cemiplimab (REGN-2810)**	IgG4	PD-1	Advanced squamous cell carcinoma (first-line alone).<br>Advanced nasal-cell carcinoma (after or not tolerance to Vismodegib).<br>Advanced NSCLC (alone in first-line in PDL-1 ≥ 50%)
**Dostarlimab (TSR-042)**	IgG4	PD-1	Advanced dMMR endometrial cancer (alone in second-line).
**Tremelimumab (CP-675)**	IgG2	CTLA-4	Advanced hepatocellular carcinoma (in first-line in combination with Durvalumab).
**Relatlimab (BMS-986016)**	IgG4	LAG-3	Advanced melanoma (in combination with Nivolumab in first-line).*

*Abbreviations: CPS combined positive score; CT chemotherapy; CTLA-4 Cytotoxic T-Lymphocyte Antigen 4; CT/RT chemotherapy plus radiotherapy; dMMR deficient DNA mismatch repair system; LAG-3 Lymphocyte-activation gene 3; NSCLC non-small-cell lung cancer; PD-1 Programmed Death 1; PD-L1 Programmed Death-ligand 1; SCLC small-cell lung cancer.

Figure S1. – Flow diagram of the study.

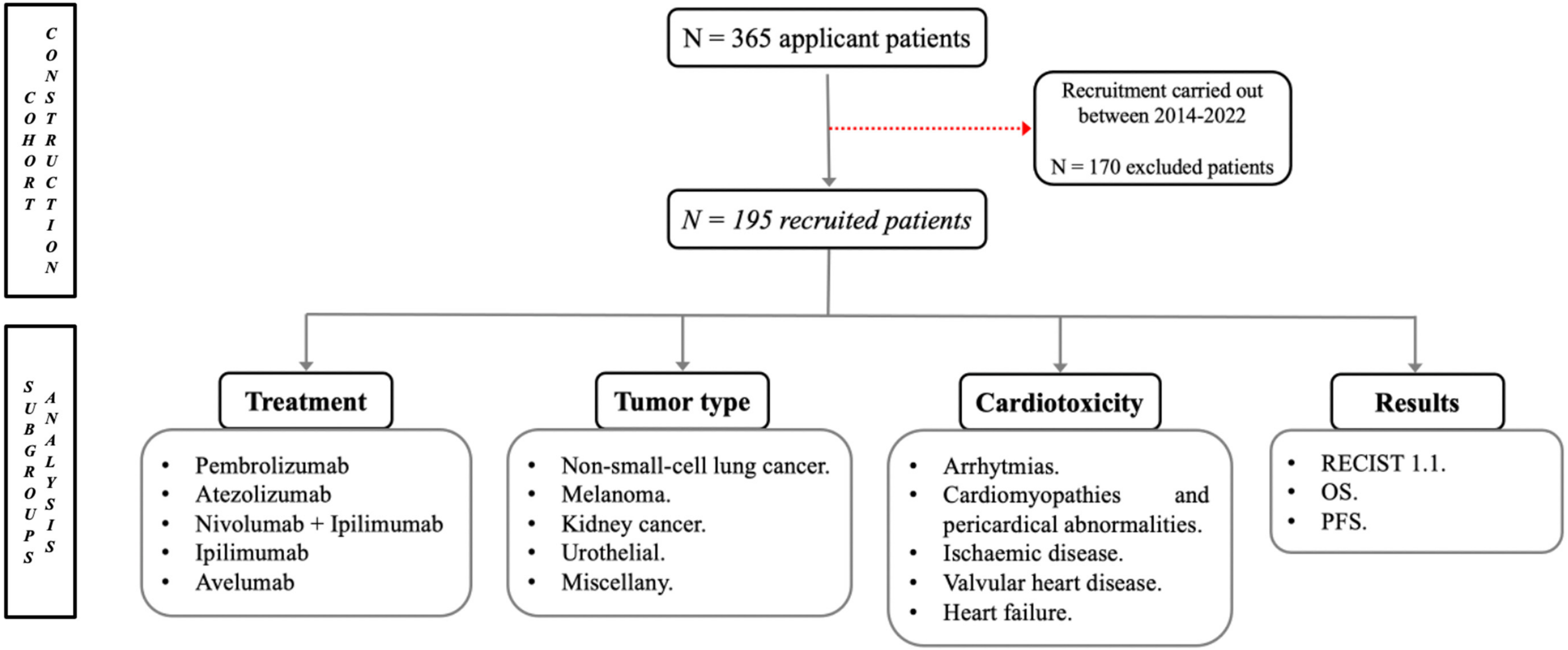

Supplement: Supplementary file 1 [file cancers-15-04293-s001.zip › cancers-2512287-supplementary.pdf]
